# Lorlatinib in pretreated ALK- or ROS1-positive lung cancer and impact of TP53 co-mutations: results from the German early access program

**DOI:** 10.1177/1758835920980558

**Published:** 2021-02-09

**Authors:** Nikolaj Frost, Petros Christopoulos, Diego Kauffmann-Guerrero, Jan Stratmann, Richard Riedel, Monica Schaefer, Jürgen Alt, Sylvia Gütz, Daniel C. Christoph, Eckart Laack, Martin Faehling, Richard Fischer, Klaus Fenchel, Sebastian Haen, Lukas Heukamp, Christian Schulz, Frank Griesinger

**Affiliations:** Department of Infectious Diseases and Respiratory Medicine, Charité Universitätsmedizin Berlin, Augustenburger Platz 1, Berlin, D-13353, Germany Corporate Member of Freie Universität Berlin, Humboldt-Universität zu Berlin, and Berlin Institute of Health, Department of Infectious Diseases and Pulmonary Medicine, Berlin, Germany; Department of Thoracic Oncology, Thoraxklinik at Heidelberg University Hospital, Heidelberg, Germany, and Translational Research Center Heidelberg, Member of the German Center for Lung Research (DZL); Division of Respiratory Medicine and Thoracic Oncology, Department of Internal Medicine V University of Munich (LMU), Thoracic Oncology Centre Munich (TOM), Comprehensive Pneumology Center Munich (CPC-M), Member of the German Center for Lung Research (DZL), Munich, Bayern, Germany; Department of Internal Medicine II, University Clinic of Frankfurt, Frankfurt, Germany; Department I of Internal Medicine, University Hospital of Cologne, Cologne, Germany; HELIOS Klinikum Emil-von-Behring, Lungenklinik Heckeshorn, Berlin, Germany; Department of Internal Medicine III (Hematology, Oncology, Pneumology), University Medical Center Mainz, Mainz, Germany; Department of Respiratory Medicine and Cardiology, Evangelisches Diakonissenkrankenhaus Leipzig, Leipzig, Germany; Department of Hematology and Oncology, Evang. Kliniken Essen-Mitte, Essen, Germany; Hämato-Onkologie Hamburg, Hamburg, Germany; Department of Cardiology, Angiology and Pneumonology, Klinikum Esslingen, Esslingen, Germany; Onkologie Dreiländereck, Lörrach, Germany; Private Practice for Hematology and Oncology, Saalfeld, Germany; Department of Hematology and Oncology, Universitätsklinikum Hamburg-Eppendorf, Hamburg, Germany; Institute for Hematopathology, Hamburg, Germany; Department of Internal Medicine II, University Hospital Regensburg, Regensburg, Germany; Department Internal Medicine-Oncology, Pius Hospital, Oldenburg, Germany

**Keywords:** ALK, brain metastases, early access program, lorlatinib, NSCLC, ROS1, TP53

## Abstract

**Introduction:**

We report on the results of the German early access program (EAP) with the third-generation ALK- and ROS1-inhibitor lorlatinib.

**Patients and Methods:**

Patients with documented treatment failure of all approved ALK/ROS1-specific therapies or with resistance mutations not covered by approved inhibitors or leptomeningeal carcinomatosis were enrolled and analyzed.

**Results:**

In total, 52 patients were included [median age 57 years (range 32–81), 54% female, 62% never smokers, 98% adenocarcinoma]; 71% and 29% were ALK- and ROS1-positive, respectively. G1202R and G2032R resistance mutations prior to treatment with lorlatinib were observed in 10 of 26 evaluable patients (39%), 11 of 39 patients showed TP53 mutations (28%). Thirty-six patients (69%) had active brain metastases (BM) and nine (17%) leptomeningeal carcinomatosis when entering the EAP. Median number of prior specific TKIs was 3 (range 1–4). Median duration of treatment, progression-free survival (PFS), response rate and time to treatment failure were 10.4 months, 8.0 months, 54% and 13.0 months. Calculated 12-, 18- and 24-months survival rates were 65, 54 and 47%, overall survival since primary diagnosis (OS2) reached 79.6 months. TP53 mutations were associated with a substantially reduced PFS (3.7 *versus* 10.8 month, HR 3.3, *p* = 0.003) and were also identified as a strong prognostic biomarker (HR for OS2 3.0 *p* = 0.02). Neither prior treatments with second-generation TKIs nor BM had a significant influence on PFS and OS.

**Conclusions:**

Our data from real-life practice demonstrate the efficacy of lorlatinib in mostly heavily pretreated patients, providing a clinically meaningful option for patients with resistance mutations not covered by other targeted therapies and those with BM or leptomeningeal carcinomatosis.

## Introduction

Rearrangements of anaplastic lymphoma kinase (ALK) or c-ros oncogene 1 (ROS1) are identified in 3–5 and 1–2% of patients with lung adenocarcinoma.^1,2^ A common pattern of clinical characteristics distinguishes these patients from the general population of non-small-cell lung cancer (NSCLC), including a younger age, a history of never or light smoking (<10 pack years), and a higher prevalence of brain metastases (BM).^3–5^ Compared with ALK-positive patients, ROS1-positive patients exhibit fewer extrathoracic and BM at first diagnosis as well as during the course of disease.^6^ Median progression-free survival (PFS) using the first-generation tyrosine kinase inhibitor (TKI) crizotinib ranges between 7.7 months in ALK-positive and 19.1 months in ROS1 patients, respectively.^7–9^ Due to a low intracranial penetration rate, the brain represents the most prominent site of progression on crizotinib treatment.^5,10–12^ Consequently, first-line treatment with more potent second-generation TKIs has become the standard of care at least for ALK-positive patients.^13^ However, the majority of patients inevitably relapses also using these newer drugs, revealing the medical need for sequential treatment options.^13,14^ Acquired mutations in the ALK or ROS1 kinase domain represent a major molecular mechanism of resistance. Lorlatinib is a potent and selective third-generation, ATP-competitive oral ALK and ROS1 kinase inhibitor, especially designed to penetrate the blood–brain barrier and to overcome known ALK and ROS1 resistance mutations. Clinical trials including ALK- and ROS1-positive patients demonstrated the efficacy of lorlatinib in subsequent lines of therapy with high intracranial response rates (RRs).^15,16^ We here report on the results of the German early access program (EAP) of lorlatinib, providing data on mostly heavily pretreated patients with ALK- and ROS1-alterated NSCLC from the daily routine.

## Patients and methods

Patients with documented treatment failure of all approved ALK/ROS1-specific therapies and ⩾2 other approved systemic therapies for metastatic NSCLC could be enrolled into the EAP from April 2017 until May 2019. Patients with documented resistance mutations not covered by other inhibitors (e.g. G1202R for ALK and G2032R for ROS1) or leptomeningeal carcinomatosis (LMC) could receive lorlatinib even without having been treated with all approved lines of therapy. Resistance testing was not mandatory prior to enrolment. LMC was defined as the combination of multifocal neurologic signs, typical radiomorphologic findings in brain/spine magnetic resonance imaging (MRI) (e.g. diffuse leptomeningeal contrast enhancement), cytologic identification of malignant cells within the cerebrospinal fluid (CSF) and/or a CSF composition compatible with LMC (e.g. high protein concentration, low glucose concentration, lymphocytic pleocytosis). Detailed inclusion and exclusion criteria were as follows.

### Inclusion criteria

1. Diagnosis:

Evidence of histologically or cytologically confirmed diagnosis of metastatic NSCLC (Stage IV, AJCC v7.0) carrying an ALK or ROS1 rearrangement as determined by locally approved tests.

2. Disease Status Requirements:

Documented treatment failure (i.e. disease progression, symptom deterioration or intolerance to therapy) of all locally approved ALK/ROS1 inhibitor therapies and any other alternative approved systemic treatments for metastatic NSCLC had to be documented as a prerequisite.

For patients with ALK-positive NSCLC:

- All approved ALK inhibitors^*^
- Plus two other approved chemotherapies or immuno-oncologic (IO) therapies

For patients with ROS1-positive NSCLC:

- Minimum crizotinib^*^ plus two other approved chemotherapies or IO therapies

^*^with the exception of documented resistance mutations not covered by other inhibitors (e.g. ALK G1202R resistance mutation).

3. Adequate Bone Marrow Function, including:
  - Absolute Neutrophil Count (ANC) ⩾1.5 × 10^9^/L;
  - Platelets ⩾100 × 10^9^/L;
  - Hemoglobin ⩾9 g/dL.
4. Adequate Pancreatic Function, including:
  - Serum total amylase ⩽1.5 × ULN
  - Serum lipase ⩽1.5 × ULN
5. Adequate Renal Function, including:
  - Serum creatinine ⩽1.5 × ULN or estimated creatinine clearance ⩾60 mL/min as calculated using the method standard for the institution.
6. Adequate Liver Function, including:
  - Total serum bilirubin ⩽1.5 × ULN;
  - Aspartate Aminotransferase (AST) and Alanine Aminotransferase (ALT) 2.5 × ULN; ⩽5.0 × ULN in the case of liver metastases.
7. Acute effects of any prior therapy resolved to baseline severity or to Common Terminology Criteria for Adverse Events (CTCAE) Grade 1, except for adverse events (AEs) not constituting any safety risk for the patient as judged by the investigator.
8. A negative serum pregnancy test for females of childbearing potential at screening.
9. Evidence of a personally signed and dated informed consent document indicating that the patient has been informed of all pertinent aspects of the “Härtefallprogramm”.

### Exclusion criteria

1. Major surgery (within 4 weeks), minor surgery (within 2 weeks), chemotherapy (within 4 weeks), radiotherapy (RT) (within 2 weeks; 48 h for palliative RT), any investigational agents (within 4 weeks), or other anti-cancer therapy (within 2 weeks; but five half-lives if known for approved TKI).
2. Clinically significant cardiovascular disease (that is, active or <3 months prior to enrolment): cerebral vascular accident/stroke, myocardial infarction, unstable angina, congestive heart failure (New York Heart Association Classification Class ⩾ II), second-degree or third-degree atrioventricular (AV) block (unless paced) or any AV block with PR >220 msec. Ongoing cardiac dysrhythmias of NCI CTCAE Grade ⩾2, uncontrolled atrial fibrillation of any grade, bradycardia defined as <50 bpm (unless patient is otherwise healthy such as long-distance runners, etc.), machine-read ECG with QTc >470 msec, or congenital long QT syndrome.
3. Predisposing characteristics for acute pancreatitis (e.g. uncontrolled hyperglycemia, current gallstone disease, alcoholism) in the last month.
4. History of extensive, disseminated, bilateral or presence of Grade 3 or 4 interstitial fibrosis or interstitial lung disease including a history of pneumonitis, hypersensitivity pneumonitis, interstitial pneumonia, interstitial lung disease, obliterative bronchiolitis and pulmonary fibrosis. Patients with history of prior radiation pneumonitis are not excluded.
5. Recent (i.e. within previous 6 months) or active suicidal ideation or behavior.
6. Concomitant use of strong or moderate CYP3A4 inhibitors, strong CYP3A4 inducers, drugs that are CYP3A4 substrates with narrow therapeutic indices:
  6.1. Current use or anticipated need for food or drugs that are known strong or moderate CYP3A4 inhibitors, including their administration within 10 days prior to the first dose of lorlatinib [i.e. strong CYP3A4 inhibitors: grapefruit juice or grapefruit/grapefruit related citrus fruits (e.g. Seville oranges, pomelos), ketoconazole, miconazole, itraconazole, voriconazole, posaconazole, clarithromycin, telithromycin, indinavir, saquinavir, ritonavir, nelfinavir, amprenavir, fosamprenavir nefazodone, lopinavir, troleandomycin, mibefradil, and conivaptan; Moderate CYP3A4 inhibitors: erythromycin, verapamil, atazanavir, delavirdine, fluconazole, darunavir, diltiazem, aprepitant, imatinib, tofisopam, ciprofloxacin, cimetidine].
  6.2. Current use or anticipated need for drugs that are known strong CYP3A4 inducers, including their administration within 12 days prior to the first dose of lorlatinib (i.e. phenobarbital, rifampin, phenytoin, carbamazepine, rifabutin, rifapentin, clevidipine, St. John’s Wort).
  6.3. Concurrent use of drugs that are CYP3A4 substrates with narrow therapeutic indices, such as astemizole, terfenadine, cisapride, pimozide, quinidine, tacrolimus, cyclosporine, sirolimus, (alfentanil and fentanyl, including transdermal patch) or ergot alkaloids (ergotamine, dihydroergotamine) is not permitted or caution is warranted. CYP2C9 or P-gp substrates with narrow therapeutic indices, sensitive CYP2B6 substrates, or strong CYP2C8 or CYP2C19 inhibitors.
7. Concurrent use of drugs that are CYP2C9 substrates with narrow therapeutic indices, such as warfarin, phenytoin or a sensitive substrate such as celecoxib is not permitted or caution is warranted.
8. Concurrent use of drugs that are sensitive CYP2B6 substrates, such as bupropion, efavirenz is not permitted or caution is warranted.
9. Current use or anticipated need for drugs that are known strong CYP2C19 inhibitors, including their administration within 12 days prior to study entry (i.e. fluconazole, fluvoxamine, ticlopidine).
10. Current use or anticipated need for drugs that are known strong CYP2C8 inhibitors, including their administration within 12 days prior to study entry (i.e. gemfibrozil).
11. Current use or anticipated need for drugs that are known P-gp substrates with a narrow therapeutic index, including their administration within 12 days prior to study entry (i.e. digoxin).
12. Breastfeeding female patients (including patients who intend to interrupt breastfeeding).
13. Known hypersensitivity to lorlatinib or any of its excipients.

Patients’ baseline demographics, tumor-specific data and outcome were collected. As RECIST-based radiologic evaluation is not routinely performed in the real-world setting, estimation of tumor response and definition of disease progression were based on the clinician’s estimates, which have nevertheless been shown to correlate reasonably well with RECIST assessments in several recent studies.^17,18^ On-treatment imaging was performed according to national guidelines^19^ and the respective local standard of care, respectively, using computed tomography scans (thorax, abdomen) and brain MRIs. Positron emission tomography scans were not routinely performed.

Survival endpoints were real-world PFS, time to treatment failure (TTF) and overall survival (OS). PFS was defined as the time in months from the first dose of lorlatinib to the first documented progression, either as radiologically confirmed progression or death, PFS2 as the time in months from the first dose of any subsequent therapy following lorlatinib until death from any cause, TTF as the time in months from the first dose of lorlatinib until loss of clinical efficacy as defined by the treating physician, OS1 as the time in months from the first dose of lorlatinib and OS2 since the date of primary diagnosis until death from any cause.

AEs were graded according to the CTCAE version 5.0. Approval was obtained from the Charité Universitätsmedizin Berlin ethics committee (approval number EA2/159/19).

## Statistical analysis

Demographics and disease data were described and compared using the Pearson Chi^2^-test, Fisher’s exact test or Mann–Whitney *U* test, according to the level of measurement. The Kaplan–Meier method was used to estimate median PFS, TTF and OS. *p*-values comparing survival curves were calculated with log-rank tests. Hazard ratios were calculated using univariate Cox-regression analysis. All analyses were performed using IBM SPSS statistics version 24 (IBM, Armonk, NY, USA). A *p*-value < 0.05 (two-tailed) was defined as statistically significant.

## Results

### Baseline characteristics

In total, 52 patients from 29 institutions were included in the analysis. Some 37 patients were ALK-positive (71.2%), 15 had a ROS1 rearrangement (28.8%). The median number of prior therapies was five (range 2–9) and three (1–5) in ALK and ROS1-positive patients, respectively. ALK-positive patients were pretreated with three (1–4) specific TKIs, and rates for crizotinib, ceritinib, alectinib and brigatinib were 94.6, 89.2, 91.9 and 35.1%, respectively. All ROS1 patients had received crizotinib and 26.7% an additional treatment with ceritinib. In ALK-positive patients, the most recent treatment regimens patients progressed on before lorlatinib were alectinib (17 patients, 45.9%), chemo-/immunotherapy (eight patients, 21.6%), ceritinib (five patients, 13.5%), brigatinib (four patients, 10.8%) and crizotinib (three patients, 8.1%). The most recent treatments in ROS1-positive patients were crizotinib (nine patients, 60.0%) and ceritinib (three patients, 20.0%). One patient each (6.7%) was treated with chemo-/immunotherapy and cabozantinib, respectively. Thirty-six patients (69.2%) had active BM at the time of enrolment and nine (25%) exhibited signs of LMC. The presence of BM was associated with a PS of ⩾2 (*p* = 0.04). All baseline characteristics are listed in Table 1.

**Table 1.** Patients’ baseline demographics in the entire cohort (left column) and in ALK and ROS1-patients separately (middle and right column).

	All patients (*n* = 52)	ALK-positive (*n* = 37)	ROS1-positive (*n* = 15)
Age, years (range)	57 (32–81)	58 (32–70)	56 (36–81)
Sex, *n* (%)			
Female	28 (53.8)	18 (48.6)	10 (66.7)
Male	24 (46.2)	19 (51.4)	5 (33.3)
Smoking history, *n* (%)			
Current smoker	4 (7.7)	3 (8.1)	1 (6.7)
Former smoker	14 (26.9)	10 (27.0)	4 (27.6)
Never smoker	32 (61.5)	22 (59.4)	10 (66.7)
Missing data	2 (3.8)	2 (5.4)	
Performance Status at primary diagnosis, *n* (%)			
0	31 (59.6)	24 (64.9)	7 (46.7)
1	16 (30.8)	10 (27.0)	6 (40.0)
2	1 (1.9)	1 (2.7)	
missing data	4 (7.7)	2 (5.4)	2 (13.3)
Histology, *n* (%)			
Adenocarcinoma	51 (98.1)	37 (100.0)	14 (93.3)
Adenosquamous carcinoma	1 (1.9)		1 (6.7)
Brain metastases at primary diagnosis, *n* (%)			
Yes	13 (25.0)	11 (29.7)	2 (13.3)
No	38 (73.1)	25 (67.7)	13 (86.7)
Missing data	1 (1.9)	1 (2.7)	
Stage at primary diagnosis			
III	6 (11.5)	4 (10.8)	2 (13.3)
IV	46 (88.5)	33 (89.2)	13 (86.7)
Prior systemic therapies, *n* (range)	4 (1–9)	5 (2–9)	3 (1–5)
Prior targeted therapies, *n* (range)	3 (1–4)	3 (1–4)	1 (1–2)
Crizotinib	50 (96.2)	35 (94.6)	15 (100.0)
Ceritinib	37 (71.2)	33 (89.2)	4 (26.7)
Alectinib	34 (65.4)	34 (91.9)	
Brigatinib	13 (25.0)	13 (35.1)	
Performance Status at enrolment, *n* (%)			
0	12 (23.1)	8 (21.6)	4 (26.7)
1	27 (51.9)	19 (51.4)	8 (53.3)
2	9 (17.3)	7 (18.9)	2 (13.3)
3	2 (3.8)	2 (5.4)	
4	2 (3.8)	1 (2.7)	1 (6.7)
Brain metastases at enrolment, *n* (%)			
Yes	36 (69.2)	26 (70.3)	10 (66.7)
No	16 (30.8)	11 (29.7)	5 (33.3)
Leptomeningeal disease at enrolment, *n* (%)			
Yes	9 (25.0)	6 (23.1)	3 (30.0)
No	27 (75.0)	20 (76.9)	7 (70.0)

### Response to lorlatinib

After a median follow-up time of 16.1 months [95% confidence interval (CI), 12.2–18.1], the median duration of treatment was 10.4 months (95% CI, 6.5–12.8), 25 patients (48.1%) still received lorlatinib. In 46 patients with documented responses (88.5%, no information: *n* = 4; response not evaluable: *n* = 2), RR was 54.3% with two complete responses (CR; 4.3%) and 23 partial responses (PR; 50.0%). The disease-control rate was 82.6%. RR according to the rearrangement were 42.4% for ALK-positive patients (*n* = 14) and 84.6% for ROS1 (*n* = 11, *p* = 0.02). RR in ALK-positive patients with two (*n* = 4), three (*n* = 15) or four (*n* = 16) previous TKIs were 25.0, 40.0 and 43.8%, respectively. In patients with prior alectinib (*n* = 34), RR was 38.3% as compared with 33.3% without (*n* = 3). In ROS1-positive patients having received crizotinib only (*n* = 10) and two previous TKIs (*n* = 5), respectively, RR were 80.0 and 60.0%. Comparing patients with BM with those without, RR were 62.5% and 35.7% (*p* = 0.09). Seven out of nine patients with LMC showed a PR (77.8%).

### Survival analysis, efficacy in brain metastases and subsequent therapies

**Figure 1.**
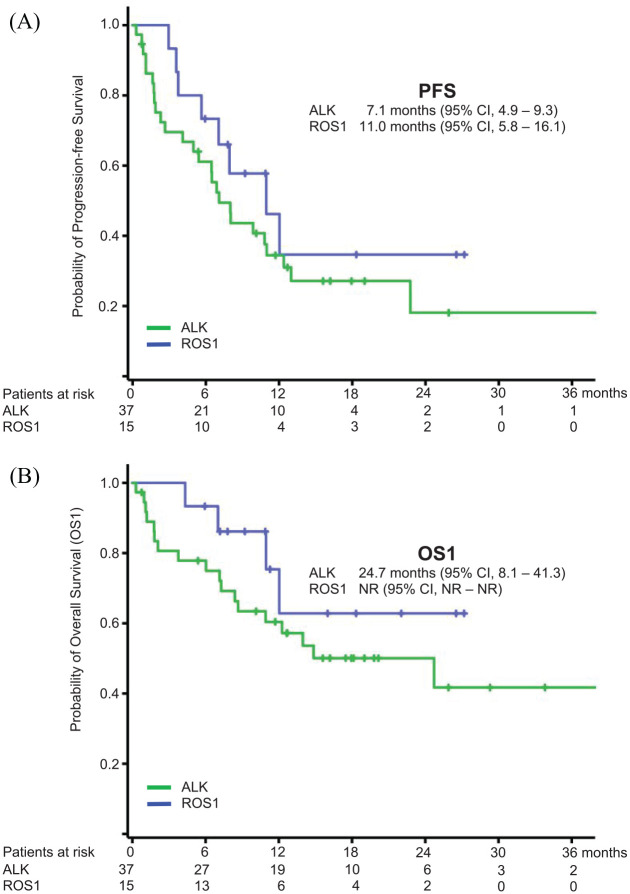
Kaplan–Meier curves for progression-free (PFS, Figure 1A) and overall survival since treatment with lorlatinib (OS1, Figure 1B) for ALK and ROS1-positive patients.

At the time of data cut-off (30 April 2020), 34 PFS events were recorded (65.4%), median PFS was 8.0 months (95% CI, 4.0–12.0, Figure 1A). 59.4% of PFS events occurred extracranially (*n* = 19), an isolated brain progression was documented in 15.6% (*n* = 5), one patient had a simultaneous extra- and intracranial progression [site of progression unknown in seven cases (21.9%)]. Ten out of 15 patients with baseline BM progressed intracranially (75%) as compared with no patient without (*p* = 0.04). The presence of baseline BM had no influence on PFS (HR 0.97, 95% CI, 0.47–1.99, *p* = 0.93).

**Figure 2.**
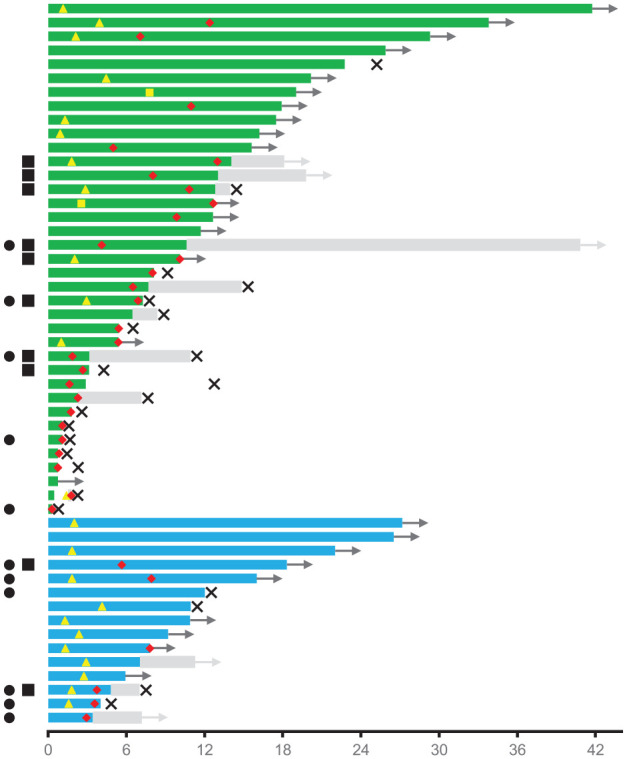
Swimmer plot for each individual patient indicating the duration of treatment with lorlatinib and subsequent therapies (time in months). Green bars (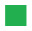) indicate ALK-positive patients on lorlatinib, blue bars (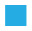) ROS1-positive patients on lorlatinib. Black arrows (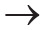) indicate ongoing treatment with lorlatinib. Yellow triangles (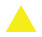) define the date of partial remission, yellow squares (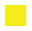) the date of complete remission. Red hashes (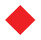) indicate the date of progressive disease. Gray bars (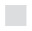) indicate subsequent therapies, gray arrows (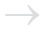) ongoing subsequent treatment(s). Black crosses (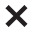) define the date of death. Black circles (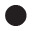) indicate TP53 mutations, black squares (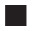) G1202R or G2032 gatekeeper mutations.

Treatment with lorlatinib was continued in 14 patients (41.2%) beyond documented progression, resulting in a TTF of 13.0 months (95% CI, 8.8–17.3). Out of 34 patients with progressive disease (PD), 12 received at least one subsequent therapy (23.1%), containing chemotherapy, immunochemotherapy or immunotherapy alone in seven, one and two patients, respectively. Seven patients were re-exposed to a specific TKI. PFS2 for subsequent treatments was 7.1 months for all patients (95% CI, 1.5–12.8) and 2.2 months for TKIs (95% CI, 1.9–2.5) *versus* not reached in patients receiving chemotherapy (*p* = 0.11). Swimmer plots visualizing the treatment with lorlatinib and subsequent therapies for each individual patient are depicted in Figure 2.

Within the indicated follow-up time 22 deaths occurred (42.3%). The estimated median OS1 reached 24.7 months [95% CI, not evaluable (NE)–NE], calculated 12-, 18- and 24-months survival rates were 64.9, 54.0 and 47.3%, respectively (Figure 1B). OS2 reached 79.6 months (95% CI, 75.1–102.1).

### Molecular characterization and identification of potential resistance mechanisms

Next-generation sequencing (NGS)-based analyses of potential tyrosine kinase resistance mutations prior to therapy with lorlatinib were performed in 26 patients (50.0%, Table 2). Substantially more ALK-positive patients underwent a re-assessment (either tissue-based or liquid biopsy) than those with ROS1 (62.2 *versus* 20.0%, *p* = 0.01). Specific mutations were identified in 15 patients (57.7%), six of whom displayed compound mutations with ⩾2 mutations. G1202R and G2032R-mutations represented the most frequent resistance pattern, detected in eight ALK (61.5%) and two ROS1-positive patients (100.0%). PFS and TTF for patients with G1202R/G2032R/without detectable mutations *versus* those with compound mutations were 6.9 (95% CI, 3.4–10.3) and 12.8 months (95% CI, 6.9–18.8) *versus* 1.9 (95% CI, 0.0–4.8) and 3.2 months (95% CI, 0.0–12.5), respectively.

**Table 2.** Tyrosine kinase resistance mutations and TP53 mutations in the entire cohort (left column) and in ALK and ROS1-patients (middle and right column).

	All patients (*n* = 52)	ALK-positive (*n* = 37)	ROS1-positive (*n* = 15)
Assessment of specific tyrosine kinase mutations (NGS), *n* (%)	26 (50.0)	23 (62.2)	3 (20.0)
Tyrosine kinase mutation^*^	15 (57.7)	13 (56.5)	2 (66.6)
V1149A		1	
C1156Y		2	
I1171N		1	
F1174V		1	
L1196M		4	
G1202R		8	
D1203N		1	
G1269A		2	
G2032R			2
No tyrosine kinase mutation	11 (42.3)	10 (43.5)	1 (33.3)
Assessment of TP53 mutations, *n* (%)	41 (78.8)	31 (83.8)	10 (66.7)
TP53 mutation	11 (26.8)	5 (16.1)	6 (60.0)
No TP53 mutation	28 (68.3)	25 (80.7)	3 (30.0)
TP53 not evaluable	2 (4.9)	1 (3.2)	1 (10.0)

* compound mutations (*n* = 6): L1196M-based: +I1171N, +F1174V, +D1203N; G1202R-based: +V1149A+L1196M, +C1156Y, +G1269A.

NGS, next-generation sequencing.

**Figure 3.**
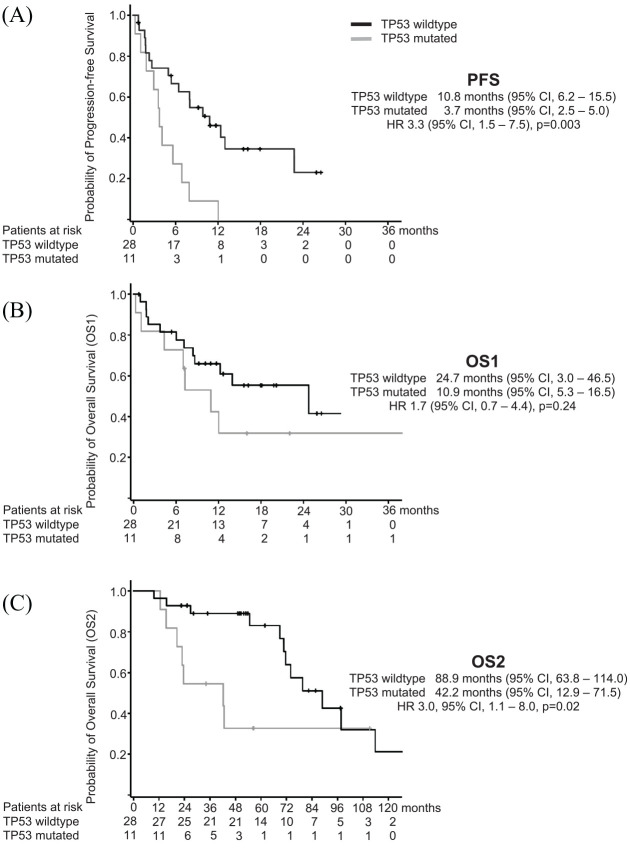
Kaplan–Meier curves for progression-free survival (PFS, Figure3A), overall survival since treatment with lorlatinib (OS1, Figure 3B) and overall survival since primary diagnosis (OS2, Figure 3C), each depending on the TP53 mutational status.

NGS-based analyses of TP53-mutations at any timepoint prior to lorlatinib were conducted in 41 patients (78.8%). Pathogenic mutations were detected in 11 patients (26.8%), occurring more frequently in ROS1- (6/10 patients, 60%) than ALK-positive patients (5/31 patients, 16.1%, *p* = 0.01). TP53 mutations were associated with a threefold decrease in PFS of 3.7 (95% CI, 2.5–5.0) *versus* 10.8 months (95% CI, 6.2–15.5, HR, 3.3, 95% CI, 1.5–7.5, *p* = 0.003, Figure 3A). By trend, survival differences were also observed for OS1 (10.9 *versus* 24.7 months, *p* = 0.24, Figure 3B). In general, TP53-mutations carried out a strongly negative prognosis throughout the entire course of disease with an OS2 of 42.2 (95% CI, 12.9–71.5) *versus* 88.9 months (95% CI, 63.8–114.0, HR 3.0, 95% CI, 1.1–8.0, *p* = 0.02, Figure 3C).

### Adverse events

AEs were reported in 37 patients (71.2%); the median number per patient was two (range 1–8; 14 patients without reported AEs, information missing *n* = 1). All AEs are listed in Table 3. The most frequent clustered AEs concerned gastrointestinal disturbances (34 patients), followed by neurological and respiratory disorders (16 and 11 patients, see footnote below Table 3 for a detailed description of all neuro-psychiatric disorders). The most common isolated AEs were hypercholesterolemia (*n* = 17) and peripheral edema (*n* = 11). The majority of AEs were mild or moderate grade (G1 or G2, 59 events, 37 patients). G3 and four events occurred in eight (nine events) and two patients (two events), respectively, concerning laboratory abnormalities in lipid metabolism and pancreatic enzymes as well as edema, central nervous system effects and pneumonitis. Grading was not reported for 21 events. Therapy with lorlatinib was discontinued due to AEs in five patients, suffering from dyspnea (G3), anasarca (G3), an increase in serum creatinine [grade not reported (NR)] and pneumonitis (G4). Psychiatric disorders leading to treatment discontinuation concerned hallucinations and persecution mania (grade NR) in two ALK-positive patients with BM and LMC.

**Table 3.** Summary of adverse events.

Event	Any grade	G1 or 2	G3	G4	Grade not reported
Patients with adverse events (AE), *n*	37	37	8	2	4
AE reported, *n*	91	59	9	2	21
AE leading to discontinuation, *n*	5	1	1	1	2
Gastrointestinal disorders	34	29	4	0	1
Diarrhea	3	3	0	0	0
Dysgeusia	2	1	0	0	1
Hypercholesterolemia	17	15	2	0	0
Hypertriglyceridemia	6	5	1	0	0
Lipase/amylase increased	4	3	1	0	0
Mucositis	1	1	0	0	0
Nausea	1	1	0	0	0
General disorders	21	13	2	0	6
Edema	11	8	2	0	1
Fatigue	2	2	0	0	0
Pruritus	1	0	0	0	1
Rash	4	1	0	0	3
Sweating	1	0	0	0	1
Weight gain	1	1	0	0	0
Neurologic disorders	16	7	1	0	8
Central nervous system effects^*^	13	5	1	0	7
Peripheral neuropathy	3	2	0	0	1
Respiratory disorders	11	5	2	1	3
Dyspnea	7	5	0	0	2
Pleural effusion	1	0	1	0	0
Pneumonia	1	0	0	0	1
Pneumonitis	2	0	1	1	0
Other disorders	9	5	0	1	3
Creatinine increased	1	0	0	0	1
Hypertension	1	1	0	0	0
Hypothyroidism	2	2	0	0	0
Myalgia	2	1	0	0	1
Thromboembolism	2	1	0	1	0
Tongue swelling	1	0	0	0	1

* Psychiatric disorders: *n* = 4 (aggressiveness, hallucinations, persecution mania, panic attack); visual defects: *n* = 3; dizziness: *n* = 3; slow speech, headache and daze: *n* = 1 each.

## Discussion

Our data from real-world treatment demonstrates the efficacy of lorlatinib in mostly heavily pretreated patients with either ALK- or ROS1-rearrangements. RR was 42.4% for ALK-positive patients (*n* = 14), comparable to a phase II study^16^ and the results from the expanded access program in Asian countries and the US.^20^ In contrast to these studies, RR was not influenced by the number of prior TKIs and was also substantially higher for ROS1-positive patients (84.6%) as compared with the respective phase I/II study.^15^

The brain represents a frequent site of progression in ALK- and ROS1-positive NSCLC, affecting nearly 60% of patients after 3 years.^21^ In the crizotinib-refractory setting, intracranial RR for alectinib, brigatinib and ceritinib ranged between 35 and 73%.^22^ According to the presence *versus* absence of BM at baseline, lorlatinib showed rates of brain progression after 12 months of 22 *versus* 9% after crizotinib, and 23 *versus* 12% after ⩾1 second generation ALK-TKI.^23^ Although BM have not been evaluated separately in our investigation, the reported RRs were higher in patients with BM (62.5% *versus* 35.7%) suggesting a high intracranial efficacy. In contrast to the published literature, the brain also represented the most frequent site of progression in patients suffering from BM when entering the EAP (75.0%), whereas no patient without BM progressed intracranially. Due to these uneven results, a clear brain-protecting effect of lorlatinib could not be identified from our dataset. LMC represents a difficult to treat manifestation of BM, mostly refractory to standard treatment and associated with a dismal prognosis. In this setting, case series suggest a high efficacy using effective targeted therapy even after treatment failure of preceding ALK TKIs.^24^ The prevalence of LMC in NSCLC in general is estimated to range between 3% and 5%^25^ and substantially increases in molecularly altered subgroups, benefiting from a longer survival due to targeted therapies. The 25% of patients with LMC in our study may represent an enriched share of patients even for this special population,^20^ showing a promising RR of 77.8%.

The median PFS reached 8.0 months and was 7.1 and 11.0 months for ALK and ROS1 patients, consistent with phase I/II studies reporting a PFS of 7.3 and 8.5 months in the ALK and ROS1 subsets, respectively.^15,16^ Treatment beyond progression was continued in almost half of the patients with PD (14 patients, 41.2%) and resulted in a meaningful increase in TTF of 13.0 months. Treatment continuation beyond PD given an ongoing clinical response nowadays represents an established option for patients with molecularly altered NSCLC and has been proven beneficial. Hence, retrospective data suggest that these patients might represent a subset with a more favorable prognosis in general.^26^

In the era of molecularly driven therapies, offering sequential targeted treatment options in ALK and ROS1-positive NSCLC, re-characterization of the tumor *via* repeated biopsies has become an established procedure and was carried out in 50% of patients in the routine setting. G1202R-solvent-front mutations are associated with clinical resistance to first and second-generation ALK TKIs but may be sensitive to lorlatinib.^27^ G2032R-mutations represent a similar situation for ROS1-patients. The available molecular data from the present investigation confirm the clinical efficacy of lorlatinib for both solvent-front mutations, whereas PFS and TTF were clearly reduced with the evidence of compound mutations. Furthermore, TP53 mutations were associated with a markedly reduced PFS of 3.7 *versus* 10.8 months. As differences in OS since primary diagnosis depending on the TP53 status were even more pronounced (42.2 *versus* 88.9 months), these alterations might represent an intrinsic mode of TKI resistance. In this line, several investigations have identified TP53 mutations, either present at diagnosis or acquired at disease progression, as a predictive as well as prognostic biomarker in ALK-positive NSCLC.^28,29^ To our knowledge, our study is the first report transferring the validity of these results also on a treatment with lorlatinib, indicating that TP53 mutations confer a negative impact on PFS and OS irrespective of the TKI.

Whether re-exposure to a frontline TKI after progression on a subsequent TKI may be beneficial remains an unresolved question. In our study, patients receiving another TKI after lorlatinib had a shorter PFS2 than those treated with chemotherapy. However, selection bias may represent a significant confounder, as patients with a subsequent TKI may have been judged not fit enough for chemotherapy and data on post-lorlatinib PS were not reported. A significant fraction of the so far identified lorlatinib-resistant compound mutations is not sensitive to earlier generation TKIs.^30–32^ Therefore, a re-exposure without a carefully performed molecular analysis suggesting sensitivity to first- or second-generation TKIs has to be regarded cautiously. Thus, patients may benefit more from chemotherapy.

The reported rates of AEs in our study were substantially smaller than those in the respective clinical trials, probably attributable to a less rigorous reporting in the daily routine. However, as patients were not treated according to a dedicated study protocol, dose modifications may have been used preemptively to avoid (more severe) AEs. It is noteworthy, that two of five patients with AE-related treatment cessation were discontinued due to psychiatric disorders, effects that have not been reported for other ALK or ROS1-TKIs. Especially BM (e.g. next to the limbic system) and LMC might predispose to psychiatric AEs. In this connection, prior brain radiotherapy and steroids administered to most patients with symptomatic BM have been described as potential trigger factors.^33^ Albeit the biologic connection with the administration of lorlatinib is uncertain, it would be highly desirable to identify patients at risk in advance, as these AEs have a substantial impact on the patients’ quality of life and might require discontinuation of a highly effective treatment.

Due to its retrospective design, this study has some limitations. ALK fusion variants, proven to impact response and survival on TKIs, were not assessed routinely and thus were not evaluable.^34,35^ On the other hand, TP53 status and molecular resistance mechanisms at the time of disease progression were assessed at all participating centers in laboratories and by NGS platforms certified by the quality management initiative of the German Society of Pathology (QuIP^®^), therefore the results can be considered homogenous and reliable. Patients were treated within the valid standard of care outside a clinical trial with varying imaging intervals, potentially biasing PFS, and imaging was not routinely performed using RECIST assessments. In this line intra- and extracranial responses were not assessed separately and are not reported from our cohort. Nonetheless and with special regard to these limitations, a growing body of evidence suggests that radiologic outcome generated from a real-life cohort may be comparable to a RECIST-defined study cohort.^17,18^ An important limitation results from the special patient characteristics of nearly every EAP. Data from real-world practice demonstrate a substantial loss of patients in the transition from one line of therapy to the other.^36^ The recently reported FLAURA trial comparing osimertinib with erlotinib in EGFR-mutant NSCLC indicated that 22% of patients with PD did not receive any second-line treatment and 46–49% had no third-line therapy.^37^ Thus, patients included into an EAP represent a clearly positively selected population and immortal-time bias should be taken into consideration with regard to OS. Results must be regarded with caution and may not be translated to a general population.

## Conclusion

Our data from a real-world setting confirm the effectiveness of lorlatinib in heavily pretreated ALK and ROS1-positive patients, in whom all treatment options are exhausted. Control of BM is promising, especially as those patients are at a high risk to develop BM during the course of their disease. Integration of the TP53 status into molecular testing strategies may provide a helpful tool in the management of disease, as patients displaying mutated TP53 have a more aggressive course of disease and may derive a clinical benefit from a closer monitoring.
